# Fine needle aspiration diagnosis of extracranial glioblastoma multiforme: Case report and review of the literature

**DOI:** 10.1186/1742-6413-2-19

**Published:** 2005-11-14

**Authors:** Stacey Schultz, Gregory S Pinsky, Nancy C Wu, Marc C Chamberlain, A Sonali Rodrigo, Sue E Martin

**Affiliations:** 1 Department of Pathology, USC/Keck School of Medicine, Los Angeles, USA; 2 Department of Medicine, USC/Keck School of Medicine, Los Angeles USA

## Abstract

**Background:**

Hitherto uncommon, the incidence of extracranial metastases of primary brain malignancies may increase, with improved treatment methods and longer patient survival. Fine needle aspiration biopsy is a simple, safe and reliable method to diagnose metastatic malignancy. It has definite advantages over tissue biopsy, which is more invasive and is of higher risk to the patient. Ours is a case of glioblastoma multiforme, which metastasized to the scalp and was diagnosed on fine needle aspiration biopsy. Only a few articles document the cytological features of extracranial glioblastoma multiforme, diagnosed by fine needle aspiration biopsy.

**Case presentation:**

We report the case of an elderly female who presented with focal neurological symptoms. She was diagnosed radiologically with an intracranial lesion in the left temporal region, which was subsequently resected. Histology revealed a glioblastoma multiforme confirmed by immunohistochemistry. The tumor recurred subsequently and the patient was treated with chemotherapy, intraoperatively. At a later stage, she presented with a scalp mass on which fine needle aspiration biopsy was performed. The cytomorphological features aided by immunohistochemistry supported a diagnosis of metastatic glioblastoma multiforme. The mass was later resected and histology confirmed the fine needle aspiration diagnosis of glioblastoma multiforme.

**Conclusion:**

Reports of extracranial metastases of primary brain tumors are few. When they do occur, the primary cause is implantation during surgery or biopsy. However, spontaneous metastases to other organs do occur rarely. We believe fine needle aspiration biopsy to be very useful in the diagnosis of metastatic glioblastoma multiforme. The ability to use a cellblock for immunohistochemical studies is greatly advantageous and helpful in differentiating this tumor, from other malignancies that can occur in the scalp. A detailed discussion of the material obtained from fine needle aspiration biopsy of metastatic glioblastoma multiforme is presented, as well as a review of previous accounts in the literature.

## Background

Extracranial metastasis of primary brain malignancies is an uncommon event [1]. With increased patient survival due to new treatment options, however, the incidence of extracranial metastases of brain malignancies may increase, as has been the case with systemic cancers. An alternative to tissue biopsy to determine whether or not extracranial spread has occurred, is fine needle aspiration (FNA) biopsy. An FNA biopsy has several advantages over tissue biopsy, including immediate evaluation of tissue adequacy, minimal risk to the patient and the possibility of multiple passes to reduce the risk of sampling error. Because of the rarity of metastases of primary brain malignancies, the cytological findings of metastatic primary brain tumors are limited to only a few accounts. A literature review produced a total of five articles pertaining to the FNA biopsy features of extracranial metastatic primary brain tumors [2-6]. Four of the articles involved gliomas (three glioblastoma multiforme [3,4,6], and one low-grade astrocytoma [5]) and one pertained to a sacrococcygeal myxopapillary ependymoma [2]. This case report is submitted to detail the cytological findings in an extracranial glioblastoma multiforme (GBM).

## Case presentation

### Clinical history

A 74-year-old female with no significant past medical history presented with complaints of headache in conjunction with speech and memory problems for approximately one month. Magnetic resonance imaging (MRI) revealed an enhancing cystic lesion in the left temporal lobe. A partial resection of the left temporal mass, revealed a malignant neoplasm on frozen section and on final pathological evaluation, a diagnosis of GBM was made. Immunohistochemical results showed positive staining for glial fibrillary acidic protein (GFAP) and no evidence of positive staining for cytokeratin. The patient received adjuvant external beam radiation therapy.

Approximately nine months later the patient returned with both speech and short-term memory problems. MRI revealed tumor recurrence in the left temporal area. Stereotactic biopsy demonstrated a necrotic tumor consistent with GBM on frozen section, a diagnosis confirmed on permanent sections. The stereotactic biopsy was followed by intraoperative injection of the chemotherapeutic agent DTI-015 [7]. Three months later, the patient returned with a 2.0 cm subcutaneous mass in the left parietal scalp, approximately six centimeters from the closest postoperative scar. Fine needle aspiration biopsy of the left parietal scalp mass revealed a GBM. The lesion was excised a month later with histological confirmation of the diagnosis. Following excision of the scalp mass, a repeat MRI documented progression of the primary tumor. The patient died of pulmonary embolism one month after excision of the scalp mass.

FNA biopsy of the palpable left parietal subcutaneous scalp mass was performed by a pathologist, using a 23 gauge needle. Four smears and one cell block were prepared. The air dried smears were stained with a modified Wright stain (Diff-Quik^®^); the 95% ethanol fixed smears were stained with the Papanicolaou stain.

### Microscopic description

**Figure 1:**
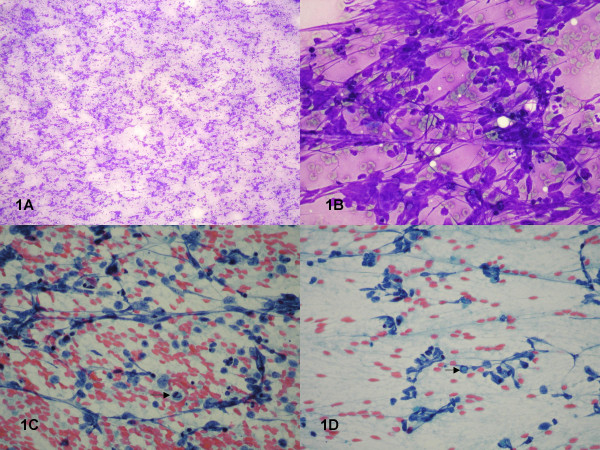
**Figure 1A**:  Smear showing abundant cellularity and necrosis (Diff-Quik ® stain, X100). **Figure 1B**:  Smear showing atypical cells in loosely cohesive clusters (Diff Quik ® stain, X400). **Figures 1C and 1D**:  Smears showing pleomorphic cells with coarsely clumped chromatin, irregular nuclear membranes, prominent nucleoli, atypical mitosis (1C, arrow), intranuclear inclusion (1D, arrow) and cellular processes (Papanicolaou stain X400)

**Figure 2:**
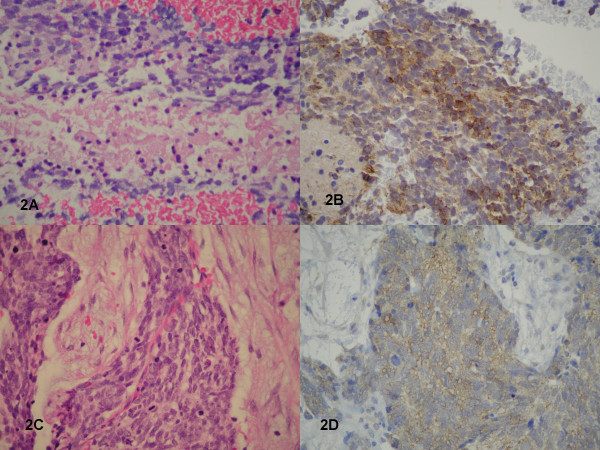
**Figure 2A**:  Cell block showing geographic necrosis (H&E stain, X400). **Figure 2B**:  Cell block showing positive staining of malignant cells for glial fibrillary acidic protein (immunostain for GFAP, X400). **Figure 2C**:  Histology of surgical resection showing malignant cells with numerous mitotic figures in a desmoplastic background (H&E stain, X400). **Figure 2D**:  Histology of surgical resection showing positive staining of malignant cells for glial fibrillary acidic protein (immunostain for GFAP, X400).

The smears were abundantly cellular with atypical cells arranged in loosely cohesive clusters, ranging from 5 to 40 cells per cluster, with overlapping nuclei (Fig. 1A–1D). Scattered atypical mitoses were also present (Fig. 1C). Morphologically, the cells were polygonal to spindle-shaped with increased nuclear to cytoplasmic ratios, moderate nuclear pleomorphism, coarsely clumped hyperchromatic chromatin, irregular nuclear membranes, and distinct nucleoli. Binucleated and multinucleated cells were also noted. A few scattered intranuclear inclusions were present (Fig. 1D). Fibrillary processes extending from the atypical cells were apparent. A tumor diathesis consisting of neutrophils, degenerated cells, necrotic debris, a few lymphocytes, and bare nuclei made up the background. The cell block showed similar findings as well as characteristic necrotic foci surrounded by palisading spindle-shaped atypical cells (geographic necrosis) (Fig. 2A). Immunohistochemically, the atypical cells displayed strong reactivity for GFAP (Fig. 2B) and vimentin with no reactivity for CD45, cytokeratin, and HMB-45, supporting the diagnosis of GBM. The histological sections from the tumor excised a month later verified the FNA findings of GBM (Fig. 2C–2D).

## Conclusion

The cytologic and immunohistochemical features of this FNA, augmented by the patient's history of GBM, led to a straightforward diagnosis in this case. In the absence of a detailed clinical history, however, a consideration of other malignant neoplasms that occur in the scalp may be necessary in the initial work up. Table 1 gives a brief differential comparing the cytological features of other malignancies that may occur in the scalp of adults.

**Table 1:** Cytologic Features of Malignant Neoplasms of the Scalp

Tumor	Cellularity	Cells	Nuclei	Nucleoli	Cytoplasm	Mitosis	Back-Ground	IHC
Metastatic GBM	Abundant, Primarily single cells, with occasional small loosely cohesive clusters	Round to oval to spindle	Pleomorphic, coarsely clumped chromatin, INCIs, occasional binucleation	Usually one, may have two	Scant, cytoplasmic processes	+ Atypical forms	Tumor diathesis	GFAP
Squamous cell carcinoma	Abundant, tight to loosely cohesive, disorderly groups, with single cells	Pleomorphic to uniform small cells, may see squamous pearls	Central location, densely hyperchromatic to irregular chromatin clumping	Inconspicuous to prominent	Moderate, dense with cytoplasmic keratinization	+ Atypical forms	Depends on grade of tumor	Keratin
Basal cell carcinoma	Large tight crowded clusters with peripheral palisading	Basaloid	Small, round to oval, hyperchromatic	Inconspicuous may be prominent	Scant	+ No atypical forms	Pink, amorphous material	Ber-EP4
Angiosarcoma	Scant to moderate, small clusters with single cells	Whorl formation, bland to pleomorphic, erytrhophagocytosis	Hyperchromatic, shallow longitudinal grooves	Prominent in high grade neoplasms	Scant to abundant, vacuolated	+ Atypical forms	Bloody, necrotic	Factor VIII, CD 31 CD 34
Melanoma	Moderate to high, loosely cohesive groups, numerous single cells	Epithelioid to spindle to pleomorphic	Eccentric location, binucleation common, few INCIs	Macronucleoli	Moderate, granular, vacuoles, melanin	+ Atypical forms	Clean to bloody, pigment-laden macrophages	S100 HMB45

Extracranial spread of GBM, although rare, is a recognized phenomenon with an estimated incidence of less than 0.5%. It usually occurs in patients with a history of a previous craniotomy or a diversionary shunt. Proposed routes of dissemination include implantation of tumor during open surgery and during stereotactic biopsy [8]. Rare cases of spontaneous metastasis without a history of biopsy or surgery have been reported in the literature [9]. The first well-documented extracranial metastasis was reported in 1928 [10]. The sites of metastasis vary with the type of brain primary and include bone, lung, liver, and lymph node. In the pediatric population, medulloblastoma is the leading tumor for extracranial metastasis. In the adult, it is malignant astrocytoma/GBM. True metastases as well as extracranial recurrences have been reported as being diagnosed by FNA biopsy [7].

In previous reports, as well as this one, the specimens procured by FNA from extracranial GBM are quite cellular consisting of malignant-appearing cells arranged in small, loosely cohesive, disorderly clusters with a predominance of single cells. Cellular size ranges from small to large with variably shaped nuclei ranging from round to oval to spindle. All descriptions consistently include marked nuclear pleomorphism with high nuclear to cytoplasmic ratios, coarsely clumped hyperchromatic chromatin, nuclear membrane irregularity, prominent single or multiple nucleoli, multinucleation and necrosis. Occasional intranuclear and cytoplasmic inclusions and rare mitoses may be seen. Scant cytoplasm is noted in all cases; however, the presence of cytoplasmic processes extending from the malignant cells creating a fibrillary background (a characteristic feature for astrocytic neoplasms) is not described in all cases. A GFAP stain may be helpful in highlighting this feature. Thick-walled capillaries with endothelial proliferation are documented in one case study [4], but this feature is absent in our case, even with immunostaining for CD34. Cell block sections do reveal the characteristic geographic necrosis typical of GBM. In addition, the cell block allows the performance of a panel of immunostains. For this reason we recommend submitting at least one FNA pass, solely for the purpose of obtaining a cell block. As in this case, the cell block may be extremely helpful in rendering a definitive FNA diagnosis of this rare extracranial tumour.

## List of abbreviations

Fine needle aspiration (FNA)

Glial fibrillary acidic protein (GFAP)

Glioblastoma multiforme (GBM)

## Competing interests

The author(s) declare that they have no competing interests.

## Authors' contributions

SS (since deceased) carried out the initial literature review and first draft of manuscript. GP assisted with preparation of the manuscript and photography. NW assisted with the cytologic evaluation and description. MC provided clinical information. SR carried out additional literature review and subsequent drafts of manuscript. SM participated in the coordination of the study and preparation of the final draft of the manuscript.
